# *In vitro* inhibition of biofilm and virulence factor production in azole-resistant strains of *Candida albicans* isolated from diabetic foot by *Artemisia vulgaris* stabilized tin (IV) oxide nanoparticles

**DOI:** 10.3389/fcimb.2023.1322778

**Published:** 2024-01-25

**Authors:** Mohammad Zubair, Fohad Mabood Husain, Marai Al-Amri, Imran Hasan, Iftekhar Hassan, Thamer Albalawi, Farha Fatima, Altaf Khan, Mohammed Arshad, Pravej Alam, Naved Ahmad, Roba Alatawy, Shamina Begum, Rashid Mir, Hisham Alshadfan, Abid Ali Ansari, Abeer Bader Abdi Al-faqir Al-Anazi

**Affiliations:** ^1^ Department of Medical Microbiology, Faculty of Medicine, University of Tabuk, Tabuk, Saudi Arabia; ^2^ Department of Food Science and Nutrition, Faculty of Food and Agricultural Sciences, King Saud University, Riyadh, Saudi Arabia; ^3^ Department of Surgery, Faculty of Medicine, University of Tabuk, Tabuk, Saudi Arabia; ^4^ Department of Chemistry, College of Science, King Saud University, Riyadh, Saudi Arabia; ^5^ Department of Zoology, College of Science, King Saud University, Riyadh, Saudi Arabia; ^6^ Department of Biology, College and Science and Humanities, Prince Sattam Bin Abdulaziz University, Alkharj, Saudi Arabia; ^7^ Department of Zoology, Aligarh Muslim University, Aligarh, India; ^8^ Department of Pharmacology, Central Research Laboratory, College of Pharmacy, King Saud University, Riyadh, Saudi Arabia; ^9^ Dental Biomedical Research Chair, College of Applied Medical Sciences, King Saud University, Riyadh, Saudi Arabia; ^10^ College of Applied Sciences, Al-Maarefa University, Riyadh, Saudi Arabia; ^11^ Department of Medical Lab Technology, Faculty of Applied Medical Sciences, University of Tabuk, Tabuk, Saudi Arabia; ^12^ Department of Clinical Biochemistry, Faculty of Medicine, University of Tabuk, Tabuk, Saudi Arabia; ^13^ Department of Biology, Faculty of Science, University of Tabuk, Tabuk, Saudi Arabia; ^14^ Faculty of Medicine, University of Tabuk, Tabuk, Saudi Arabia

**Keywords:** *Artemisia vulgaris*, tin oxide nanoparticles, Candida albicans, biofilm, drug resistance, diabetic foot

## Abstract

The advent of nanotechnology has been instrumental in the development of new drugs with novel targets. Recently, metallic nanoparticles have emerged as potential candidates to combat the threat of drug-resistant infections. Diabetic foot ulcers (DFUs) are one of the dreadful complications of diabetes mellitus due to the colonization of numerous drug-resistant pathogenic microbes leading to biofilm formation. Biofilms are difficult to treat due to limited penetration and non-specificity of drugs. Therefore, in the current investigation, SnO_2_ nanoparticles were biosynthesized using Artemisia vulgaris (AvTO-NPs) as a stabilizing agent and were characterized using ultraviolet–visible (UV–vis) spectroscopy, Fourier transform infrared spectroscopy (FT-IR), X-ray diffraction (XRD), scanning electron microscopy (SEM), and energy-dispersive X-ray spectroscopy (EDX). Furthermore, the efficacy of AvTO-NPs against biofilms and virulence factors of drug-resistant Candida albicans strains isolated from DFUs was assessed. AvTO-NPs displayed minimum inhibitory concentrations (MICs) ranging from 1 mg/mL to 2 mg/mL against four strains of C. albicans. AvTO-NPs significantly inhibited biofilm formation by 54.8%–87%, germ tube formation by 72%–90%, cell surface hydrophobicity by 68.2%–82.8%, and exopolysaccharide (EPS) production by 69%–86.3% in the test strains at respective 1/2xMIC. Biosynthesized NPs were effective in disrupting established mature biofilms of test strains significantly. Elevated levels of reactive oxygen species (ROS) generation in the AvTO-NPs-treated C. albicans could be the possible cause of cell death leading to biofilm inhibition. The useful insights of the present study could be exploited in the current line of treatment to mitigate the threat of biofilm-related persistent DFUs and expedite wound healing.

## 1 Introduction

Diabetes mellitus is associated with a variety of complications, among which diabetic foot ulcers (DFUs) are the most prevalent. DFUs manifest as open sores or wounds in the foot, resulting from the secondary effects of diabetes mellitus (DM) (Zubair and Ahmad, 2019). A DFU is a lesion that typically arises in the plantar region or digits of the foot as a result of repeated microtrauma and mechanical stress. DFUs may arise due to insufficient glycemic regulation, peripheral vascular affliction, or suboptimal foot hygiene. DFUs can happen at any age; however, they are most common in those with diabetes mellitus who are over 45. Type 2 diabetes currently affects over 405.6 million adults worldwide, and by 2030, that number is expected to rise to more than 510.8 million (Oguntibeju, 2019). In 2015 , the International Diabetes Federation estimated that DFU affected 9.1–26.1 million people having diabetes. Increased DFU prevalence has been attributed to the increased longevity of the patients with diabetes and enhanced prevalence of diabetes (Wada et al., 2023). Furthermore, DFU is a prevalent etiology of osteomyelitis and amputations affecting the lower extremities. The causes of DFUs are multifaceted. Potential contributing variables include low blood sugar levels, calluses, foot deformities, excessively tight footwear, underlying peripheral neuropathy, poor circulation, and dry skin. Neuropathy affects approximately 60% of diabetics and eventually leads to foot ulcers (Everett and Mathioudakis, 2018). Various risk factors, including but not limited to advanced age, infections, inadequate glycemic control, diabetic neuropathy, smoking, peripheral vascular disorders, ischemia, previous foot ulceration, amputation, and poor personal hygiene, are known to contribute to the development and progression of diabetic foot ulcers (Ezhilarasu et al., 2020). Furthermore, the threat of antimicrobial resistance (AMR) is a major public health concern that has been implicated in various reports conducted on DFUs (Wada et al., 2023). The incidence of multi-drug resistant organisms in DFUs is reported to be high (Rastogi et al., 2017). These ulcers, which can arise in any part of the body but are particularly common in the distal area of the lower leg, may result from microbial invasion, leading to infection and decay, and ultimately culminating in lower limb amputation. Although the rates of healing failure and mortality are influenced by both genetic and environmental factors, wound infection is also a significant contributor. Nonetheless, the challenge of distinguishing between commensal, opportunistic, and pathogenic microorganisms complicate the administration of effective treatments for such infections. Therefore, comprehending the identity of the “usual suspects” expected to be part of the skin microflora can serve as a valuable aid in unraveling this puzzle.

Fungal infections, despite their insidious nature, are often undervalued. A staggering 300 million individuals worldwide are deemed to be at exceptionally high risk for such infections, with 25 million facing a correspondingly high risk of mortality (Bongomin et al., 2017). From asymptomatic to mild skin infections to major invasive infections, fungal infections can range in clinical severity. Numerous investigations have reported that patients afflicted with diabetes manifest a proliferation of multiple microbial pathogens. Diverse scholarly works have indicated that fungal prevalence in diabetic patients varies from 7.0% to 17.38%, with Candida species, Aspergillus species, Fusarium species, Rhodotorula species, and Trichosporon species being the most recurring fungal species (Arun et al., 2019). In 2010, a preliminary investigation was conducted with the primary objective of detecting fungal infections in wounds of patients suffering from diabetes. The results of this study indicated that, in almost 30% of the cases, fungal infections were present; Candida spp. was found to be the most widespread type of infection, followed by members belonging to the Aspergillus and Trichosporon genera (Chellan et al., 2010).

The formation of biofilm is a significant stage in the pathophysiology of diabetic foot ulcers (DFUs). It plays a crucial function in both the progression and chronicity of the lesion and in the emergence of antibiotic resistance, rendering wound treatment a challenging prospect (Das et al., 2023). Infections often initiate with a disruption in the cutaneous barrier, frequently in an area of mechanical or thermal injury or ulceration. The definition of infection entails the intrusion of microorganisms and their proliferation within host tissues, leading to an activation of inflammatory responses. Subsequently, this is succeeded by the deterioration of tissues (Pitocco et al., 2019). Candida albicans is one of the most common opportunistic human pathogens that causes candidiasis. Candidiasis is responsible for mortality in various nosocomial and opportunistically abysmal persistent infections (Su et al., 2018). One reason for the fatality associated with C. albicans-related infections is the ability of the pathogen to form calcitrant biofilms. Approximately 100,000 deaths have been reported with infections initiated by biofilms (Atriwal et al., 2021). Many studies have reported the prevalence of Candida spp. in diabetic foot ulcers (Rasoulpoor et al., 2021). Although enough epidemiological data are available on the biofilms in DFU, there is a big gap in the understanding of intervention of these biofilms. The present antimicrobial intervention has become ineffective in the management of foot infections and the mitigation of amputations. Consequently, an imperative exists to identify alternative measures to avert and regulate biofilm-producing multidrug-resistant pathogens.

In recent times, nanotechnology has expeditiously surfaced as a domain of considerable interest for researchers engaged in drug development. There is a persistent endeavor to fabricate newfangled metallic and metal oxide particles at nanoscale dimensions, which bear paramount significance in the realm of medicine (Husain et al., 2022). Bio-fabrication of nanoparticles (NPs) is a cost-effective, expeditious, and more efficient approach as compared to the conventional chemical pathway. The scalability of this method to larger scales is facile, and the methodology is less arduous. Since this process employs a green approach, the use of hazardous chemicals is avoided, thereby enhancing the biocompatibility of the NPs. As a result, these NPs can be utilized in diverse biomedical and pharmaceutical applications (Chastain et al., 2019).

Among the metallic nanoparticles, tin oxide nanoparticle (SnO_2_ NPs) have attracted interest of the scientific community, as they possess many novel properties such as high chemical stability, high transparency, and low electrical sheet resistance (Bhawna et al., 2020). Green synthesized SnO_2_ NPs have been reported for antibacterial [16,17], antifungal [16], antibiofilm [17], anticancer [16], antitumor [16], and antioxidant activities (Al-Shabib et al., 2018; Khan et al., 2018). Moreover, nanoparticles of tin have been used as sensors in curtailing air pollution and in the detection of gases domestically and in the industries (Vaezi and Sadrnezhaad, 2007). Thus, it is envisaged that phyto-synthesized SnO_2_ NPs can mitigate the threat of drug-resistant biofilms formed by pathogenic C. albicans associated with DFUs. This is probably the first study investigating the effect of biosynthesized SnO_2_ NPs on biofilm forming drug-resistant C. albicans isolated from diabetic foot ulcers.

Artemisia vulgaris L. (family; Asteraceae) is a medicinal plant that has been exploited in the treatment of diabetes, depression, anxiety, stress, uterine cancer, malarial fever, stomach, and ophthalmic diseases in various parts of the world (Rehman et al., 2023). Owing to the pharmaceutical importance of A. vulgaris, we have performed synthesis of SnO_2_ NPs using the extract of A. vulgaris as a stabilizing agent to assess its potential in reducing the biofilm formed by azole-resistant C. albicans isolated from DFUs.

## 2 Materials and methods

### 2.1 Preparation of the extract

Freshly collected leaves of A. vulgaris were washed thoroughly with double distilled (DD) water, cut in small pieces, and dried under shade. Later, the dried leaves were ground to powder, and approximately 20 g of this powder was boiled (80°CC) in 100 mL of water for 1 h. Post heating, the extract was purified thorough Whatman filter paper and stored at 4°CC for further use as stabilizing agent in biosynthesis of SnO_2_ NPs.

### 2.2 Phyto-mediated synthesis of SnO_2_ NPs

Stored A. vulgaris extract was used as the stabilizing agent in biosynthesis of SnO_2_ NPs. Briefly, 30 mL of the Av extract was added drop wise in 50 mL aqueous solution of 0.05M tin chloride di-hydrate (SnCl_2_. 2H_2_O) with continuous stirring at 80°CC for 3 h. The light-yellow colored solution turned to brown color after 30 min of heating. Then, the mixture was cooled and centrifuged at 6,000 rpm for 30 min duration. Subsequently, the residue was collected and washed with absolute ethanol, followed by DD water and finally dried on hot plate. Furthermore, dried powder was calcinated inside a muffle furnace at 160–260°CC for 3 days, and the sample was labeled as AvTO-NPs.

### 2.3 Characterization of biosynthesized AvTO-NPs

Biosynthesized AvTO-NPs were characterized by UV-visible spectroscopy, FT-IR, XRD, SEM, and EDX. UV-vis spectra of AvTO-NPs was recorded using a Shimadzu UV-1800 spectrophotometer. KBr pellet method was used to obtain FT-IR spectra of the biosynthesized NPs using a Thermo Nicolet 380 spectrometer. The formation of crystal phase and unit cell of the synthesized AvTO-NPs was determined using an X-ray diffractometer (Ultima IV Rigaku Corporation, Japan). The morphology of the SnO_2_ NPs was characterized by a JEOL JSM 6510 LV (Tokyo, Japan) scanning electron microscope, and elemental analysis was performed using Oxford INCAx-sight EDAX coupled with SEM.

### 2.4 Patients and definitions

A total of 28 C. albicans isolates were collected from microbiology laboratories, which were isolated from diabetic foot patients between August 2021 and January 2023 from different hospitals in Tabuk, KSA. An infection of candidemia was defined when there is at least one Candida spp. from the sample collected from a diabetic foot ulcer patient. There must be 30 days gap for double sampling from the same patient; otherwise, multiple Candida spp. were excluded. This study was approved by the Local Research Ethics Committee (LREC) of the University of Tabuk, Tabuk, KSA, with approval no. UT-191-59-2022 under the aegis of National Committee of Bioethics (NCBE) of Kingdom of Saudi Arabia, and informed consent was obtained.

### 2.5 Identification of *Candida* species

Fungal isolates were macro- and microscopically identified based on colony morphology on media. Gram’s staining and germ tube test were performed on mucoid yeast-like growth and followed by urease test (WHO, 2009). In a suspected C.andida sp. with budding yeast-like positive Gram reaction, we performed the germ tube test (Limper, 2010). CHROM agar Candida (HiMedia, Mumbai, India) were used to differentiate C. albicans and non-albicans species morphologically by the appearance of growth type and color and sugar fermentation and sugar assimilation test. Manufacturer instruction guidelines were used to identify Candida species: C. albicans (Green). Then, C. dubliniensis and C. albicans both are germ tube positive, and C. albicans was further confirmed based on their ability to grow at 45°C (WHO, 2009; Daef et al., 2014).

### 2.6 Antifungal susceptibility test

A standard method of Clinical and Laboratory Standards Institute (CLSI) M44 for antifungal susceptibility testing was used (Limbago, 2001). Briefly, the inoculum suspension was prepared in 5 mL of sterile saline with a turbidity equivalent to 0.5 McFarland Standard. A sterile swab was dipped in inoculum and evenly spread onto 2% glucose and 5 μg/mL methylene blue supplemented Mueller–Hinton agar. We have used the following antifungal drugs in this study: fluconazole (10 μg), ketoconazole (10 μg), clotrimazole (10 μg), and amphotericin-B (10 μg). After inoculation of the test sample, plates were incubated using the manufacturer’s interpretation criteria. C. albicans ATCC 90028 was used as control for identification and antifungal susceptibility test.

### 2.7 Minimum inhibitory concentration

MIC was determined according to the recommendations proposed by the Clinical and Laboratory Standards Institute (CLSI) M27-A3 and M27-S4 documents (Ramage et al., 2001). AMB (0.002–32 µg/mL), FLZ (0.002–32 µg/mL), ITZ (0.002–32 µg/mL), KTZ (0.002–32 µg/mL), and AMB (0.002–32 µg/mL) were obtained as a ring disk, and tests were performed as per CLSI recommendations and manufacturer’s instruction.

### 2.8 Biofilm formation by microtiter plate method

The biofilm assay was done in 96-well microtiter plates using crystal violet as the staining dye as described elsewhere (Marak and Dhanashree, 2018).

### 2.9 EPS inhibition method

The EPS production by Candida strains was assessed using the method developed by Pavlova et al. (2009), with minor changes. Candida strains were grown in 50 mL of SDB at sub-MICs of test agents and centrifuged at 6,000 rpm for 30 min. Following that, 30 mL of ethanol was added to the supernatant, which was allowed to precipitate EPS at 4°C for 24 h. The precipitate was rinsed in ethanol before being centrifuged again under the same conditions. The precipitate was separated on filter paper and dried for 2 h at 50°C. After drying, the filter paper was weighed again. The increase in filter paper dry weight correlates to the amount of EPS produced. There was also an untreated control group. The experiment was carried out in triplicate. The mean weight of precipitated EPS was measured, and the % reduction in EPS generation over the untreated control was determined.

### 2.10 Germ tube method

The effect of biosynthesized AvTO-NPs on germ tube development was investigated using a modified Mackenzie approach (Mackenzie, 1962). In brief, 10 mL of culture was injected with 2 mL of sterile pooled sheep serum containing sub-MICs of AvTO-NPs. Smears of each culture were prepared after 3 h of incubation. As a control, sheep serum without NPs was used. A total of 50 cells were chosen at random from each smear, and the number of cells with germ tubes was counted. If the germ tube was at least twice the length of the cell, the cell was deemed germ tube positive.

### 2.11 Cell surface hydrophobicity

Hydrophobicity was assessed utilizing the hexadecane method, as reported previously (Rosenberg et al., 1980). Specifically, glass tubes were loaded with 1 mL of bacteria (OD_530 =_ 1.0) and supplemented with 100 μL of hexadecane (Sigma, St. Louis, MO). The mixtures underwent vigorous vortexing for 2 min, followed by an incubation period of 10 min at ambient temperature to allow for phase separation. The OD530 of the lower, aqueous phase was subsequently measured. In select cases, bovine testicular hyaluronidase (Sigma, St. Louis, MO) was used to treat the bacteria at a concentration of 2 Mg/mL at 37°C for 15 min prior to testing for adhesion to hexadecane. The percentage of hydrophobicity was determined by the following formula: % hydrophobicity = [1 − (OD530 after vortexing/OD530 before vortexing)/×100.

### 2.12 Inhibition of preformed biofilms

The biofilm of the test strains was allowed to grow for 24 h in microtiter plates. Post-incubation, non-adherent cells were removed by washing; new growth medium with or without sub-MICs of AvTO-NPs was added to each well and incubated statically at 37°CC for 24 h. Unattached cells were washed away, and adhering cells were subjected to crystal violet staining. Readings were taken at 585 nm as described earlier (Hasan et al., 2019).

### 2.13 ROS generation

ROS produced under effect of AvTO-NPs in test strains was detected using 2,7-dichlorofluorescein diacetate (DCFH-DA) dye. Test cultures were incubated with the probe dye (5 µM) at 37°CC for 4 h, and the supernatant was collected. ROS generation in the supernatant was measured at an excitation wavelength of 485 nm and emission wavelength of 525 nm (Perveen et al., 2021).

A short in vivo study was conducted to check the suitability of usage of the nanoparticles in rat animal model.

### 2.14 Animal husbandry

A total of 18 Swiss albino male rats (110 ± 20 g, 6–8 weeks old) were procured from the Animal House of the Department of Zoology (KSU, Riyadh, Saudi Arabia). They were kept in specially assigned cages in the treatment room in the Departmental Animal House (Department of Zoology, KSU, Riyadh). All the animals were kept for 10 days for acclimatization before starting the treatment with regular rat feed and fresh tap water ad libitum. Finally, the rats were separated into three treatment groups (n = 6) as follows:

Group I: Control treated with saline only;

Group II: A single dose of carbon tetrachloride (CCl_4_) at 1 mL/kg body weight [31] (Al-Tamimi et al., 2021);

Group III: Tin oxide nanoparticles at a dose of 5 mg/kg body weight daily for a week.

### 2.15 Administration of nanoparticles

Freshly prepared NPs were prepared in saline and vortexed before their administration into every animal intraperitoneally by a 1-mL insulin syringe (BD Science, USA). Animals were under strict monitoring to assess any distress and activity.

### 2.16 Preparation of *s*amples

The animals were killed on the same day after the completion of the treatment for sample collection. The Departmental Ethical Committee approved the study (Department of Zoology, KSU). The blood samples were collected in vacuum tubes (BD Science, San Jose, CA, USA) and further centrifuged (Eppendorf, Germany) to retrieve the serum samples at 1,200×g and stored at −25°C until further analysis.

### 2.17 Assessment of renal function markers

In the present study, urea and creatinine were chosen for the assessment of the functionality of the target organ. Commercial kits were used to measure all the parameters, either by linear diagnostic kits (Amposta, Spain) and kits (Quimica Clinica Aplicada S. A., Amposta, Spain), following the manufacturer’s instructions.

### 2.18 Assessment liver function markers

Aspartate transaminase (AST), alanine transaminase (ALT), and gamma-glutamyl transferase (GGT) were selected to assess liver function. Both the parameters were measured by commercial kits ((Quimica Clinica Aplicada S.A., Amposta, Spain) following the manufacturer’s instructions.

### 2.19 Statistical analysis

GraphPad Prism 5 software analyzed the data, including one-way ANOVA analysis with Tukey’s post-hoc multiple comparison test. A p-value< 0.05 was chosen as statistically significant in the present study. The asterisk marks *** were used to show significance difference from the negative control (CN^−^, group I) at<0.001, while the asterisk marks ### were used to show significance difference from the positive control (CN^+^, group II) at<0.001.

## 3 Results

### 3.1 Characterization of green synthesized SnO_2_ nanoparticles

**Figure 1:**
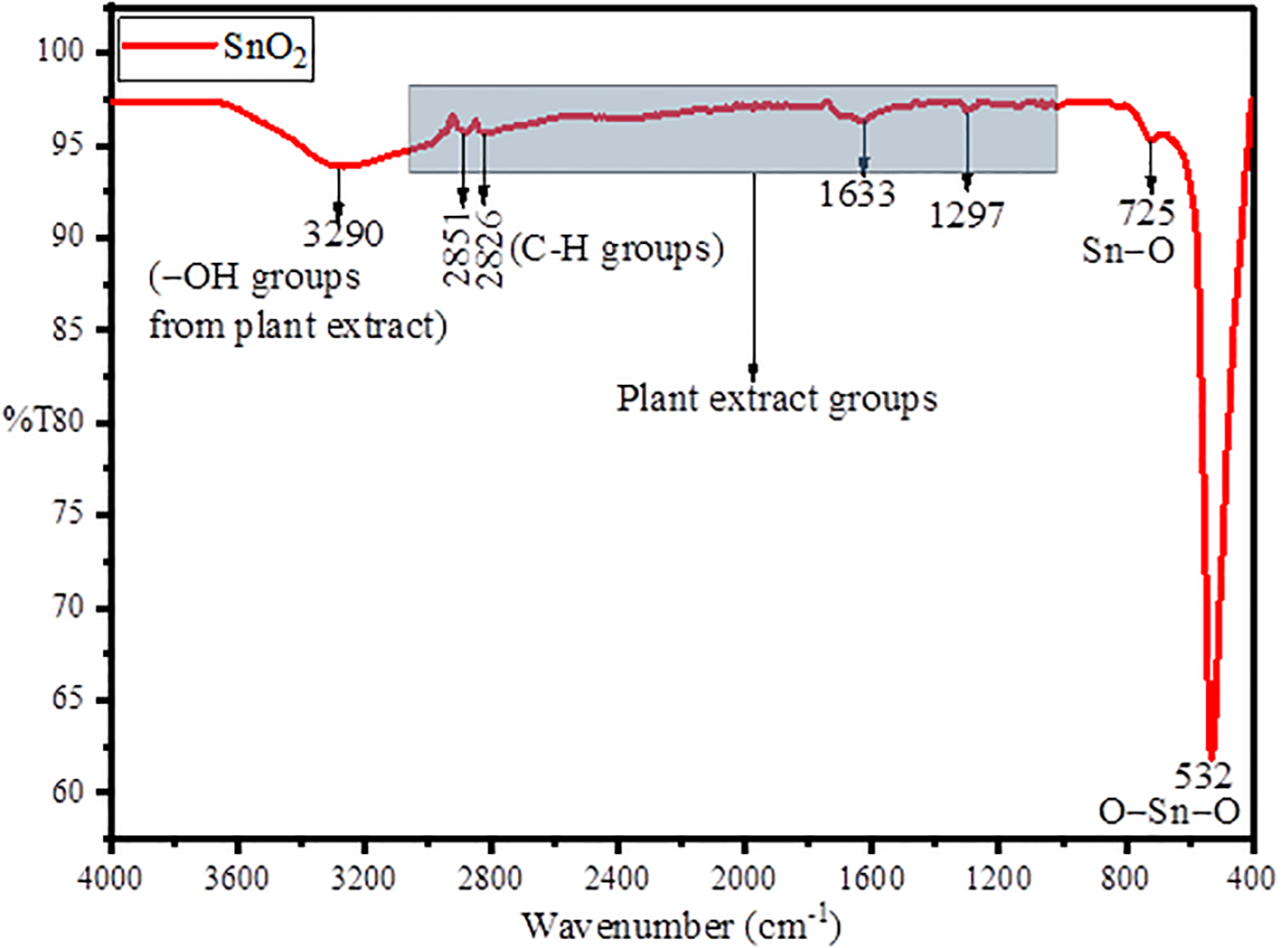
FTIR spectra of green synthesized AvTO-NPs using *A. vulgaris* extract.

**Figure 1** represents the FT-IR spectra for the green synthesized SnO_2_ NPs in which the peak at 3,290 and 1,633 cm^−1^ belongs to the stretching and bending vibrations of attached –OH groups from plan extract. The peaks at 2,851 and 2,826 cm^–1^ belongs to stretching vibrations of aliphatic C–H groups (Gomathi et al., 2021). The peak at 725 cm^–1^ belongs to surface oxygen of Sn–O, while the intense peak at 532 cm^–1^ asymmetric vibrations of O–Sn–O bond (Li and Kamali, 2022). The FT-IR results suggests the formation of SnO_2_ NPs through green synthesis using plant extract and simultaneous stabilization through –OH groups of the plant extract (Shanmugasundaram et al., 2013).

**Figure 2:**
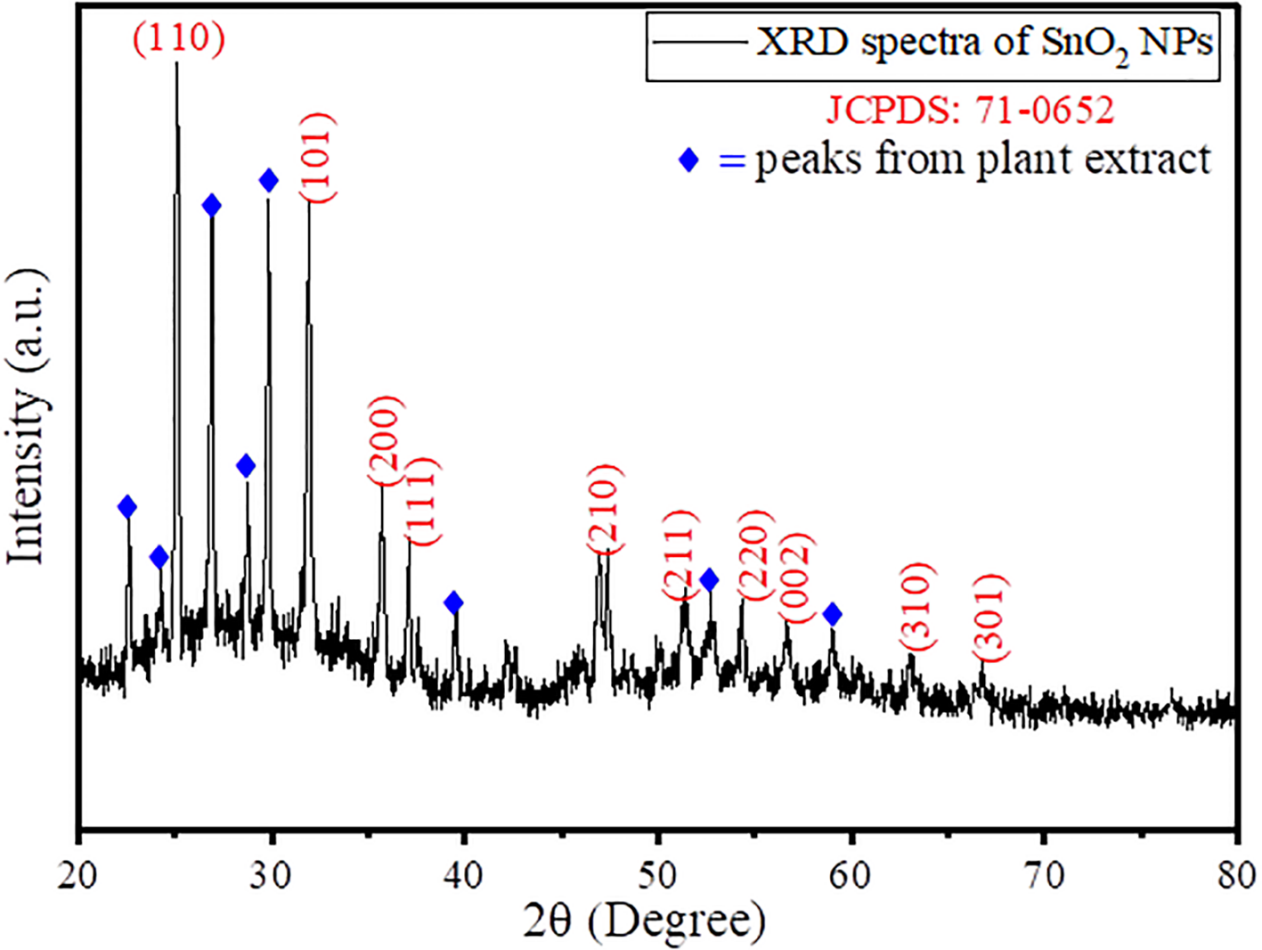
XRD spectra of green synthesized AvTO-NPs.


Furthermore, to evaluate the formation of crystal phase and unit cell, X-ray diffraction technique was taken into consideration, and the results obtained are given in **Figure 2**. The obtained XRD spectra represented the characteristic peaks of SnO_2_ NPs at 2θ values of 25.15°C, 31.85°C, 35.61°C, 37.09°C, 46.85°C, 51.39°C, 54.26°C, 56.62°C, 62.98°C, and 66.74°C belonging to Miller hkl plane as (110), (101), (200), (111), (210), (211), (220), (002), (310), and (301) in simulation with JCPDS No. 71-0652, suggesting a tetragonal rutile SnO_2_ structure (Vázquez-López et al., 2020). The other peaks that appeared in the XRD spectra belong to the plant extract, suggesting the formation of a plant extract functionalized or stabilized SnO_2_ nanoparticles. Furthermore, the average crystallite size was calculated by Debye–Scherer formula given as Equation (1) (Wang et al., 2020);

(1) $$\text{D}=\frac{\text{k}\times \text{λ}}{\text{β}\times \text{Cosθ}}$$

where D is the average crystallite size, β is the FWHM, λ is the X-ray wavelength (Cu Kα = 0.1546 nm), θ is the Bragg diffraction angle, and k is a shape factor that is in use at 0.9. Using Equation (1), the average crystallite size was found to be 48.76 nm. In the literature, the average particle size for SnO_2_ NPs was found to be in the range of 6–30 nm (Chakraborty et al., 2020; Fatimah et al., 2022). Thus, the increase in particle size clearly suggests the immobilization of a layer of plant extract metabolites around the surface of the SnO_2_ NPs. The crystallite size represents the dimension of a coherent diffracting domain within a material. The variance between the particle size and crystallites size originates from the existence of polycrystalline aggregates. The lattice mismatch resulting from the addition of plant extract primarily contributes to deviations in lattice strain (ε), which is given by Equation (2):


(2) $$\varepsilon =\frac{\beta \cos\theta }{4}$$

where θ is the diffraction angle, and β is the full width half maxima (FWHM) in radians. Using Equation (2), the value of lattice strain for the synthesized AvTO-NPs was found to be 0.046, which is quite high, suggesting that the increase in the number of nucleation sites of SnO_2_ resulted in higher grain size as observed by Debye–Scherer formula and TEM analysis.

**Figure 3:**
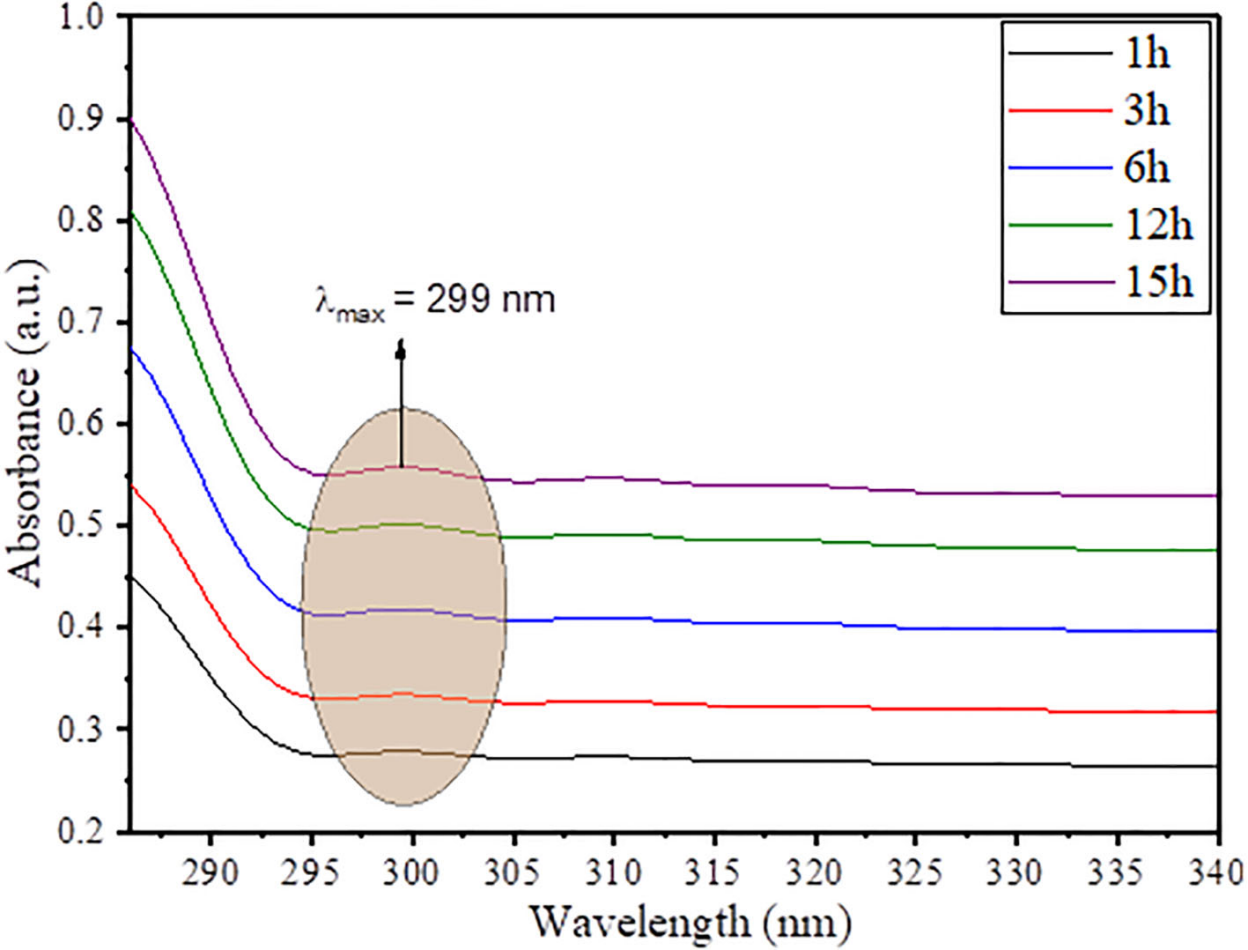
Time-dependent UV-vis spectra of AvTO-NPs.

The optical properties and simultaneous growth in nucleation of SnO_2_ NPs in the vicinity of plant extract was observed through ultraviolet visible spectroscopy. The UV spectra was taken during the synthesis of SnO_2_ NPs in a way to observe the growth in nanoparticle concentration, and the results obtained are given in **Figure 3**. As the time increases from 1 h to 15 h, there is a gradual increase in absorbance value of the reaction mixture, which suggests the gradual increase in population of SnO_2_ NPs in the vicinity of plant extract. The UV spectra exhibited a very small peak approximately 299 nm, which is due to surface plasmon resonance (SPR) effect of SnO_2_ NPs, and since the peaks are not too much sharp, it suggests that the synthesized SnO_2_ NPs are bigger in size as supported by XRD analysis and morphological analysis (Inderan et al., 2015; Fatimah et al, 2020). Moreover, from the literature, the characteristic peak for pristine SnO_2_ NPs is found approximately 345–365 nm. Thus, a blue shift of approximately 44–65 nm is due to surface functionalization of SnO_2_ NPs by plant extract polyphenolic groups (Osuntokun et al., 2017 *).*

**Figure 4:**
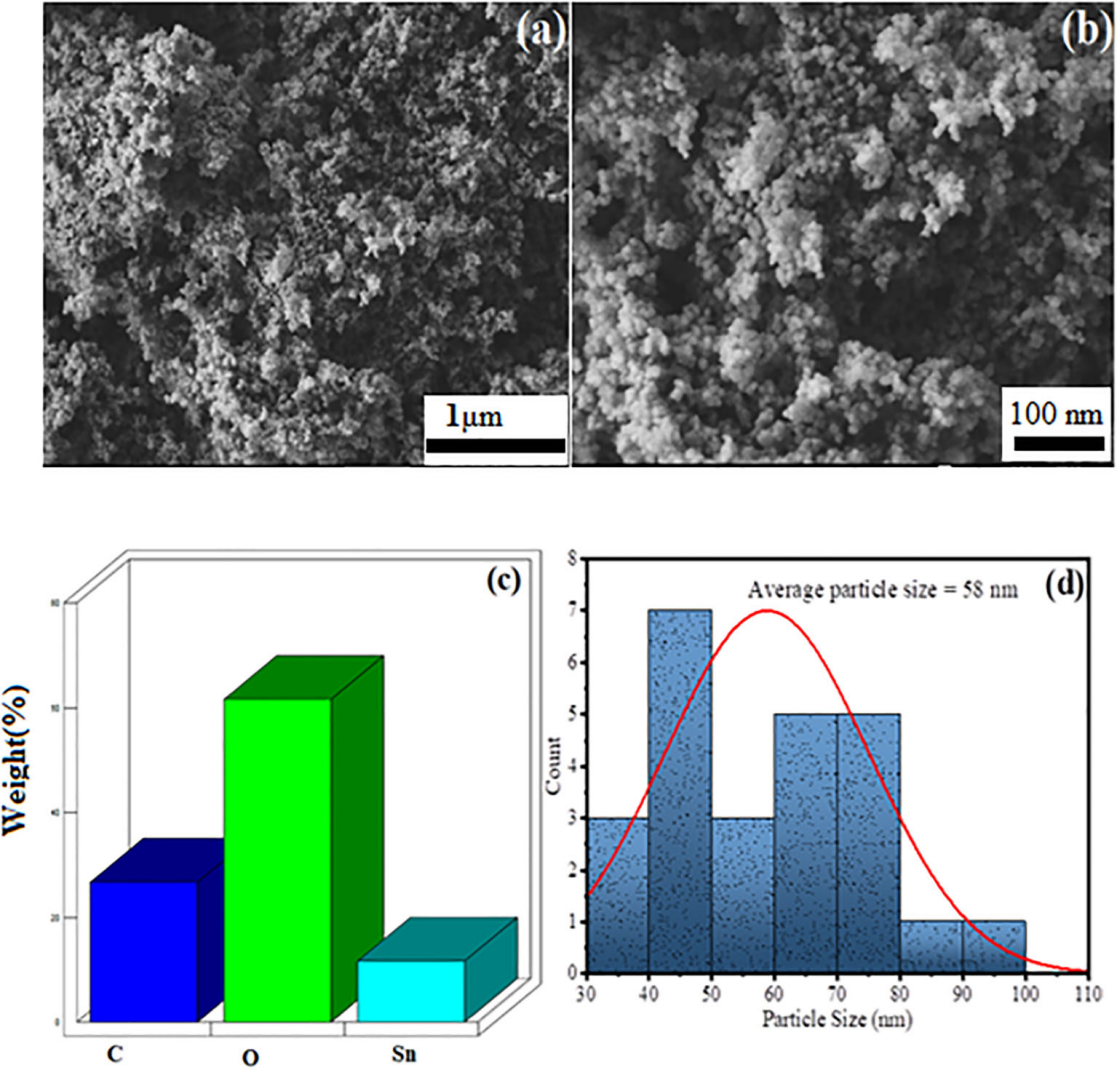
SEM image of AvTO-NPs at **(A)** 15 K, **(B)** 30 K magnification range, **(C)** EDX spectra, and **(D)** Gaussian distribution profile for average particle size distribution.

The morphological studies of the green synthesized AvTO NPs was done using a scanning electron microscope (SEM) in association with energy-dispersive X-ray (EDX) to assess the chemical composition. The obtained results are given in **Figure 4** in which **Figures 4A, B** are the SEM image of green synthesized SnO_2_ NPs at 15 and 30K magnification range. **Figure 4A** represents a porous surface, which, on further magnification, was observed as an array of spherical-shaped particles (**Figure 4B**). **Figure 4C** represents the EDX spectra of green synthesized SnO_2_ NPs, which confirms the presence of C, O, and Sn in the material as C (30.59%), O (62.27%), and Sn (7.14%). Statistical Gaussian distribution analysis was utilized to calculate the average particle size of the nanoparticles using ImageJ software, and the obtained results are given in **Figure 4D**, which shows an average particle size of green synthesized SnO_2_ NPs as 58 nm, which is also found in close agreement with the Scherer crystallite size. The high value of particle size is due to immobilization and stabilization of SnO_2_ NPs through polyphenolic groups of plant extract.

**Figure 5:**
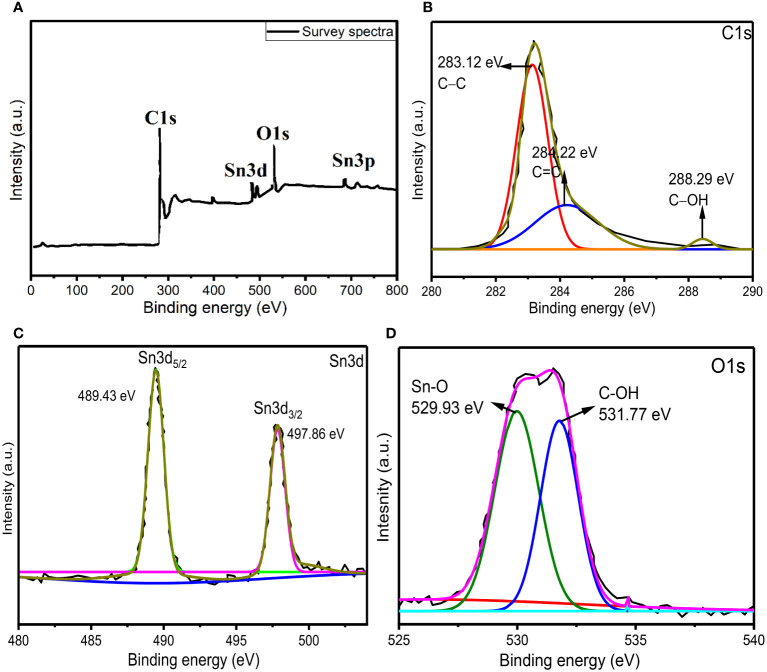
**(A)** XPS survey spectra of AvTO-NPs (**B–D**) core-level high-resolution spectra of C1s, Sn3d, and O1s with fitting and deconvolution with Gaussian distribution.

In order to further confirm the formation of AvTO-NPs, X-ray photoelectron spectroscopy (XPS) was taken into consideration, which tells about the chemical state and oxidation number of individual elements present in the material. **Figure 5A** shows the survey spectra of AvTO-NPs, which confirms the presence of C, O, and Sn in the material. **Figure 5B** represents the core-level high-resolution spectra of C1s with fitting and deconvolution with Gaussian distribution. The spectra exhibited three peaks at binding energy values of 283.12 eV, 284.22 eV, and 288.29 eV belonging to the sp3 (C−C), sp2 (C=C), and C−OH bonds from the plant extract (Eltaweil et al., 2022). **Figure 5C** represents the deconvoluted spectra for Sn3d exhibiting two peaks at 489.43 eV and 497.67 eV associated with Sn3d_5/2_ and Sn3d_3/2_ state of Sn (IV) ions, which confirms that Sn (II) ions have been oxidized to Sn (IV) ions to form SnO_2_ NPs (Kwoka et al., 2005; Flak et al., 2013). There is no detection of Sn (0) or Sn (II). **Figure 5D** shows the deconvoluted spectra of O1s, which is distribute in two peaks at 529.93 eV due to Sn (IV)−O bonds and 531.77 eV due to presence of C–OH bonds from plant extract (Siva Kumar et al., 2019; Liu et al., 2021). Consequently, these peaks appear due to interaction between functional groups of plant extract and SnO_2_, which confirms the formation of AvTO-NPs. The XPS study is consistent with the literature reports on SnO_2_ (Bonu et al., 2015; Lu et al., 2019; Peiris et al., 2022).

### 3.2 Microbiological studies

Among 122 patients with diabetic foot, 81 showed only bacterial growth, 21 showed bacterial and fungal both, only 7 patients reported solo fungal growth, and no growth was reported in 13 patients’ samples. In total, we have received 28 C. albicans. In our study, 43.9% prevalence was reported, which is in accordance with the study conducted by Kumar et al. (2016). In another study, 27.9% prevalence was also reported by Garg et al. (2008) with 76.6% Candida spp. in deep tissue wounds. It is warranted that the deep tissue sampling might affect this high prevalence of Candida spp. It is also essential to determine the complete spectrum of microbial infections of diabetic foot ulcer. The isolated C. albicans showed high resistance against amphotericin B (42.8%) followed by 21.4% resistance towards itraconazole (**Supplementary Table S1**).

### 3.3 Biofilm pattern of *Candida albicans* from DFU

The formation of biofilm by Candida is the most important virulence factor and also one of the leading causes of fungal persistence in wound causing significant clinical and economic burden to the patient who is suffering from diabetes. Among the 28 C. albicans, 18(64.2%) were biofilm producers (4 strong, 9 intermediate, and 5 weak), whereas 10 (35.7%) C. albicans showed no biofilm formation. Four strong biofilm producers C. albicans were selected for further studies (**Supplementary Table S2**).

### 3.4 MIC pattern of biofilm positive *Candida* sp.

The strong biofilm-producing C. albicans showed the highest MIC value towards fluconazole followed by ketoconazole, itraconazole, and amphotericin B as represented in **Table 1**. In addition, the average MIC values of strong biofilm is quite high compared with intermediate and week biofilm producers. Furthermore, biofilm producers demonstrated higher resistance towards the tested antifungals as compared to non-biofilm producers.

**Table 1:** MIC pattern among biofilm producers.

Isolates	Amphotericin B (0.002–32 µg/mL)	Itraconazole (0.002–32 µg/mL)	Fluconazole (0.002–32 µg/mL)	Ketoconazole (0.002–32 µg/mL)
Strong biofilm producers				
CA-KF1	4	8	64	4
CA-KF2	16	8	16	4
CA-KF3	8	16	32	32
CA-KF4	4	4	32	16
**Average MIC VALUE**	**8**	**9**	**36**	**14**
Intermediate biofilm producers				
CA-KF18	1.25	0.256	2	0.128
CA-KF06	1	0.5	2	0.25
CA-KF05	1.75	0.5	4	0.256
CA-KF07	1.75	0.25	8	1
CA-KF13	1.5	0.5	8	0.50
CA-KF14	1.25	0.25	8	0.25
CA-KF15	1.75	0.25	4	1
CA-KF16	1.25	0.25	2	0.50
CA-KF17	1.0	0.50	2	0.50
**Average MIC VALUE**	**1.389**	**0.362**	**4.444**	**0.487**
Weak biofilm producers				
CA-KF10	0.064	0.064	0.50	0.016
CA-KF08	0.016	0.008	0.25	0.008
CA-KF09	0.064	0.004	0.128	0.004
CA-KF11	0.008	0.064	0.064	0.002
CA-KF12	0.256	0.008	0.25	0.004
**Average MIC value**	**0.082**	**0.030**	**0.238**	**0.007**

Values in bold indicate average MIC values.

### 3.5 MIC determination against AvTO-NPs

Anticandidal potential of the biosynthesized AvTO-NPs against strong biofilm forming C. albicans strains was determined in terms of MIC. AvTO-NPs demonstrated MIC values of 2 mg/mL against isolated strains CA-KF2 and CA-KF4, while MIC of 1 mg/mL was recorded against CA-KF1 and CA-KF3 strains. Slightly higher MIC values of 8 mg/mL against C. albicans have been reported previously with biocompatible SnO_2_ nanoparticles (Rehman et al., 2019). This variation in the MICs could be the result of internal tolerance levels used strains, shape, and size of the SnO_2_ nanoparticle, and the method was used to determine the MICs. Furthermore, concentrations below MICs (sub-MICs) were used for biofilm and virulence assays.

### 3.6 Inhibition of biofilm formation

The existence of microbes in the state of biofilm is an important factor that contributes in delayed healing of wounds (Andrews, 2001). Pathogens are almost thousand times more resistant to antimicrobials in biofilms as compared to their free-living forms (Antić et al., 2012). Biofilm is constituted by a matrix (exopolymeric material) that envelopes the microbial cells and acts as a barrier to the action of drugs and host immune system. In DFUs, soft tissues and bones are most vulnerable to the formation of biofilm and biofilms are considered as one of the major reasons for the delayed healing of the ulcers (Zubair et al., 2021). Thus, it is imperative to assess the effect of AvTO-NPs against biofilm forming C. albicans isolated from DFUs.

**Figure 6:**
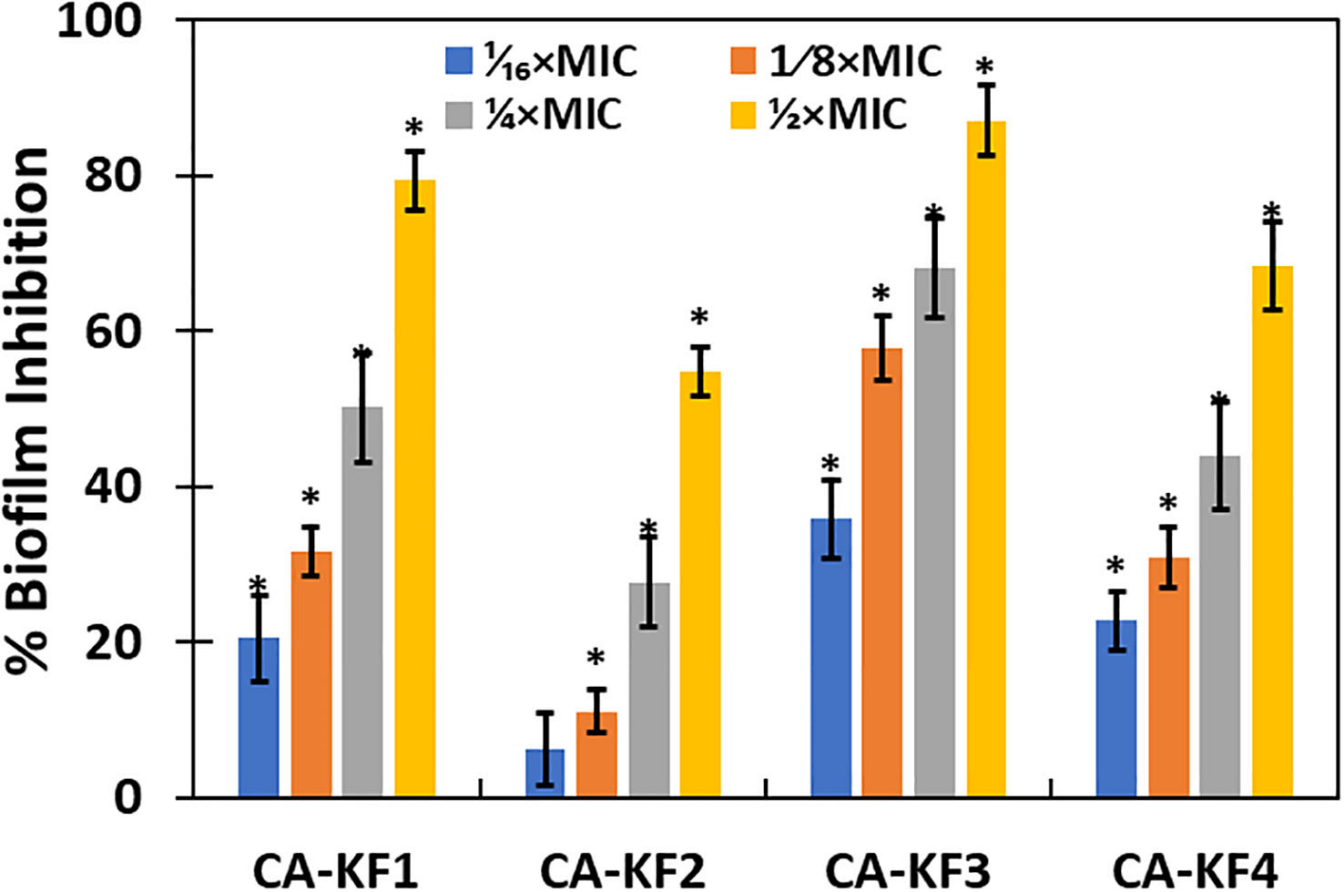
Inhibition of biofilm formation of *C. albicans* by AvTO-NPs. Data are presented as average of three replicates and error bars depicting standard deviation. * denotes significance p ≥ 0.05.

The effect of AvTO-NPs at sub-inhibitory concentrations (1/16xMIC-1/2xMIC) was evaluated against four biofilms forming strains of C. albicans isolated from DFUs. The findings of the biofilm inhibition are depicted in **Figure 6**. AvTO-NPs exerted a dose-dependent effect on biofilm formation in all the four test Candida strains. The maximum inhibition of 87.03% was observed against CA-KF3 followed by 79.3% (CA-KF1), 68.3% (CA-KF4), and 54.8% (CA-KF2) at respective 1/2xMICs of AvTO-NPs. This significant reduction in the formation of C. albicans biofilm by sub-MICs of AvTO-NPs is an important result bearing in mind the role of biofilms in development of drug resistance among pathogens and subsequent delayed healing of DFUs. Our results find support from the report on ZnO nanoparticles inhibiting biofilm formation by C. albicans isolated from urinary catheters. ZnO NPs at 50 μg/mL concentration inhibited biofilm formation in 20 fluconazole resistant C. albicans isolates (Hosseini et al., 2018). In another recent study, a chitosan/gelatin/polyvinyl alcohol xerogel film containing Thymus pubescens essential oil was synthesized to assess its antimicrobial potential in wounds. The formulation demonstrated significant antimicrobial potential against pathogens and reduced biofilm formation in C. albicans by more than 80% (Karami et al., 2023). This is probably the first report demonstrating the inhibition of biofilm formed by C. albicans isolated from DFUs by phyto-synthesized SnO_2_ NPs.

### 3.7 Biofilm inhibition on glass coverslip

**Figure 7:**
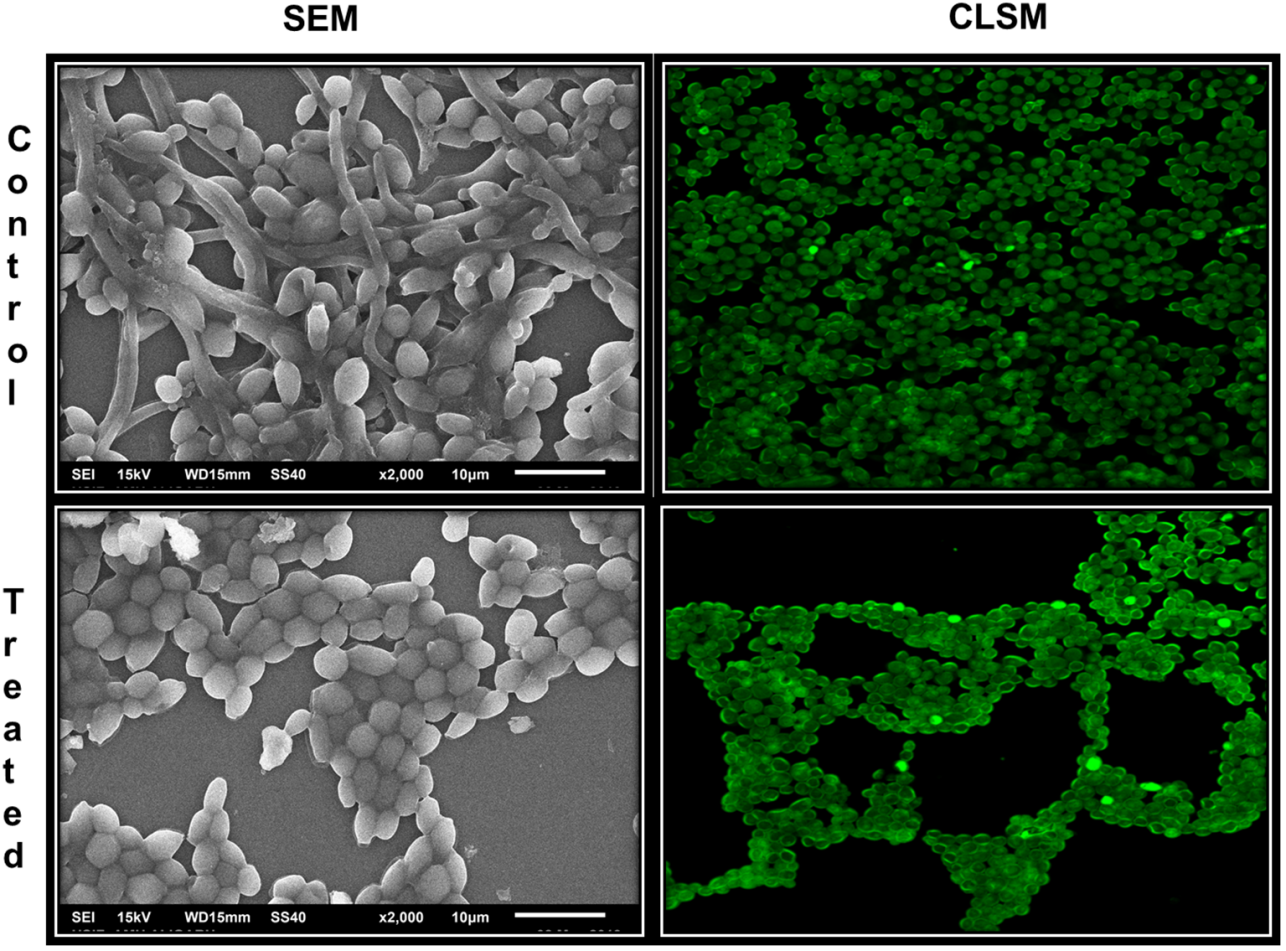
Microscopic images of biofilms of *C. albicans* treated and untreated with AvTO-NPs grown on glass cover slips.

Inhibition of biofilm formation was further analyzed on glass surface employing scanning electron microscopy (SEM) and confocal laser scanning electron microscopy (CLSM). As evident from **Figure 7**, SEM images of AvTO-NPs treated and untreated C. albicans shows changes in the biofilm architecture. Untreated yeast biofilms cells are observed to be smooth, normal, and clustered with hyphal formation. On the contrary, reduced aggregation and decreased adhesion can be observed in the NPs-treated cells. CLSM images of untreated yeast cells clearly demonstrate a thick mat-like aggregation of biofilm cells, whereas scattered cells with disturbed biofilm architecture could be observed in AvTO-NPs-treated cells (**Figure 6**).

### 3.8 Effect on germ tube formation

The formation of mycelia is an important step in the virulence and biofilm formation of C. albicans, as the mycelial form is more invasive as compared to the yeast form. Mycelial form can easily breach the mucosal barrier causing infections and represents the main virulence function associated with candidiasis. Furthermore, this filamentation is vital for the formation of strong calcitrant biofilms that cause persistent drug-resistant infections (Romo et al., 2017). However, fluconazole, the most effective antifungal used by clinicians, has demonstrated insignificant effect on germ tube formation. Thus, it is essential to halt this yeast to hyphal transformation in order to prevent the formation of biofilm by C. albicans.

**Figure 8:**
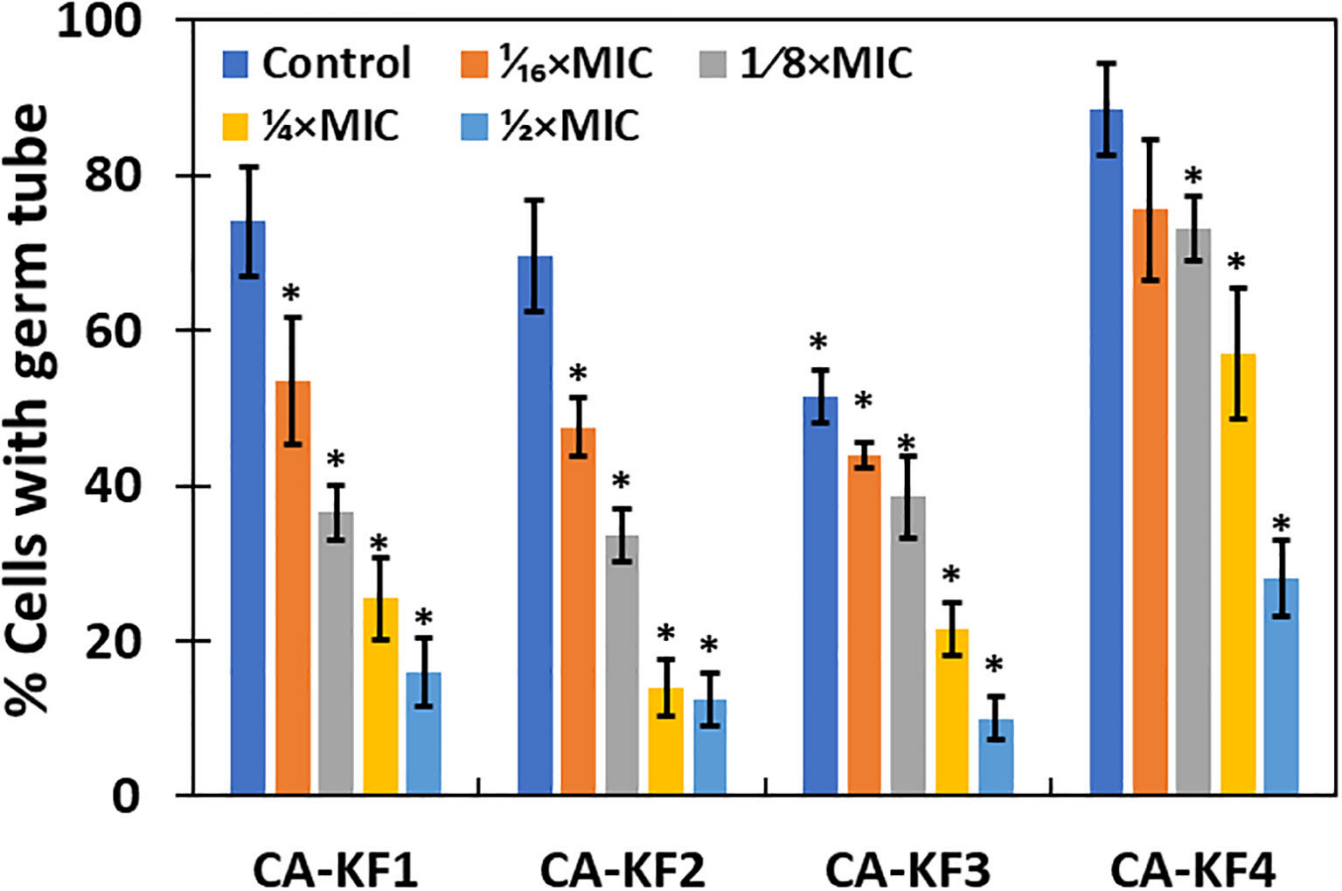
Inhibition of germ tube formation in *C. albicans* by AvTO-NPs. Data are presented as average of three replicates and error bars depicting standard deviation. * denotes significance p ≥ 0.05.

Since germ tube formation is a key virulence function associated with biofilm formation and pathogenesis of C. albicans, the effect of the sub-MICs of AvTO-NPs was assessed on the germ tube formation in isolated strains of C. albicans. As shown in **Figure 8**, significant reductions in germ tube formation was observed at concentrations ranging from 1/16xMIC to 1/2xMIC of AvTO-NPs against all the test C. albicans strains. At 1/2xMICs of the AvTO-NPs, 84%, 87.5%, 90%, and 72% reduction in the cells with germ tube was recorded in CA-KF1, CA-KF2, CA-KF3, and CA-KF4, respectively. At the lowest tested concentration (1/16xMIC), cells bearing germ tube decreased significantly (p< 0.05) by 25.5%–56% in all the test fungal strains. This is an important finding considering the importance of germ tube formation in the development of Candida biofilms. To the best of our knowledge, this is the first report on the inhibition of germ tube formation by biogenic SnO_2_ NPs. Our results are in accordance with those reported with nanoparticles of silver and zinc oxide where notable reduction of 95% and 86.4% in germ tube formation was observed (Jalal et al., 2018; Alshaikh et al., 2023).

### 3.9 Effect on cell surface hydrophobicity

**Figure 9:**
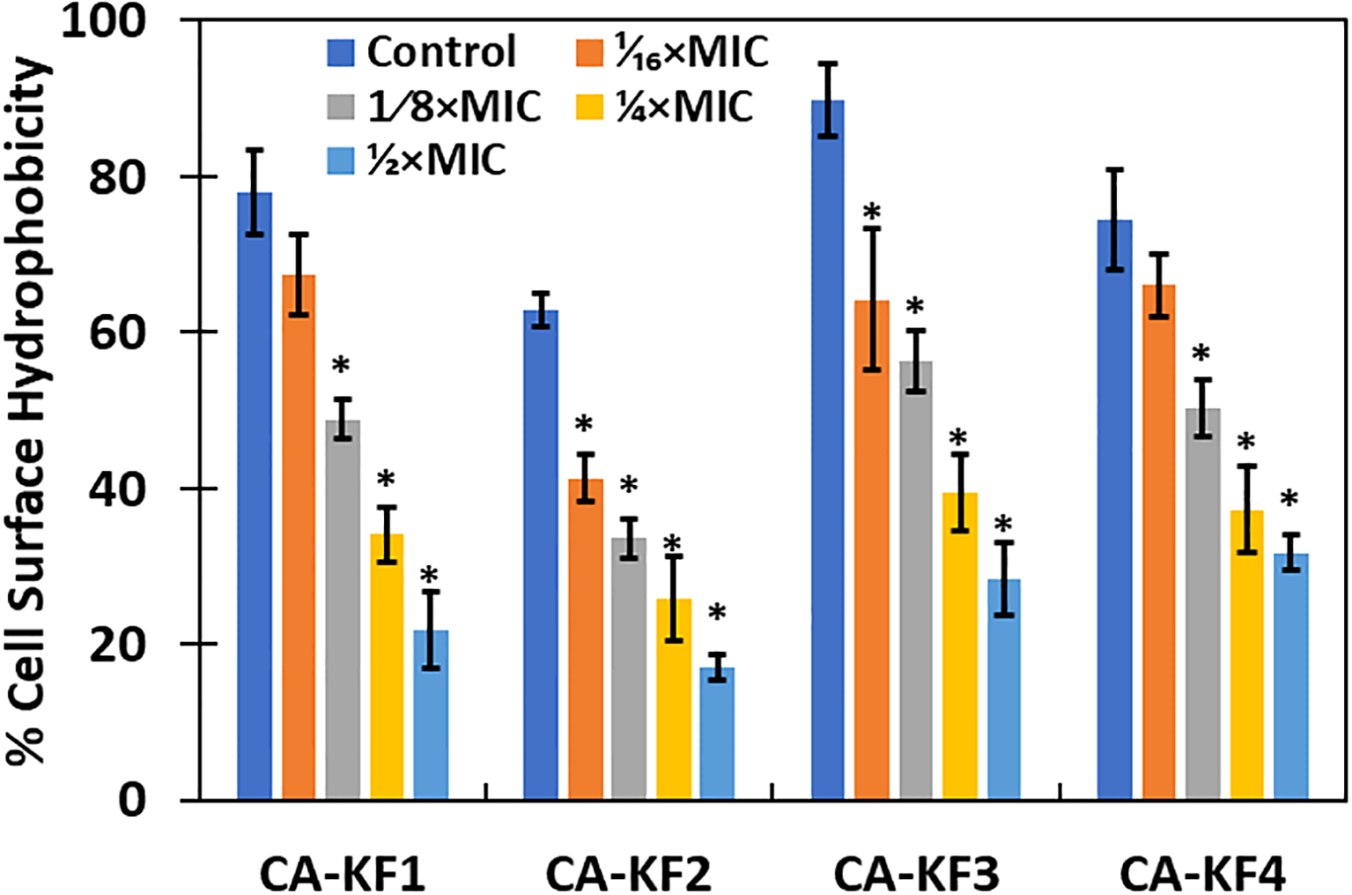
Inhibition of cell surface hydrophobicity in AvTO-NPs-treated cells. Data are presented as average of three replicates and error bars depicting standard deviation. * denotes significance p ≥ 0.05.

Hydrophobicity index of C. albicans is vital in the attachment of cells during biofilm formation. Hydrophobic cells are more virulent, as they adhere more strongly to the surface and are resistant to phagocytosis (Khan et al., 2014). In this regard, it is vital to assess the effect of sub-MICs of AvTO-NPs on the cell surface hydrophobicity (CSH) of isolated C. albicans strains. As evident from **Figure 9**, CSH was affected in varying capacity upon treatment with respective sub-MICs of AvTO-NPs. Synthesized NPs at 1/16xMIC–1/2xMIC were significantly (p< 0.05) effective in decreasing CSH in almost all the four test Candida strains. A concentration-dependent effect of the AvTO-NPs was observed. Untreated strains CA-KF1, CA-KF2, CA-KF3, and CA-KF4 demonstrated 78%, 62.8%, 89.9%, and 74.4% CSH, respectively, whereas, upon treatment with 1/2xMIC, CSH decreased to 21.9%, 17.1%, 28.3%, and 31.7% in CA-KF1, CA-KF2, CA-KF3, and CA-KF4, respectively. Results of the CSH assay clearly showed that the AvTO-NPs-treated cells had significantly decreased surface hydrophobicity as compared to the untreated cells. Reduced CSH in AvTO-NPs-treated cells is indicative of the reduced fungal colonization and hence impaired biofilm formation. To the best of our knowledge, this is the first report on the inhibition of cell surface hydrophobicity by biogenic SnO_2_ NPs.

### 3.10 Effect on EPS

**Figure 10:**
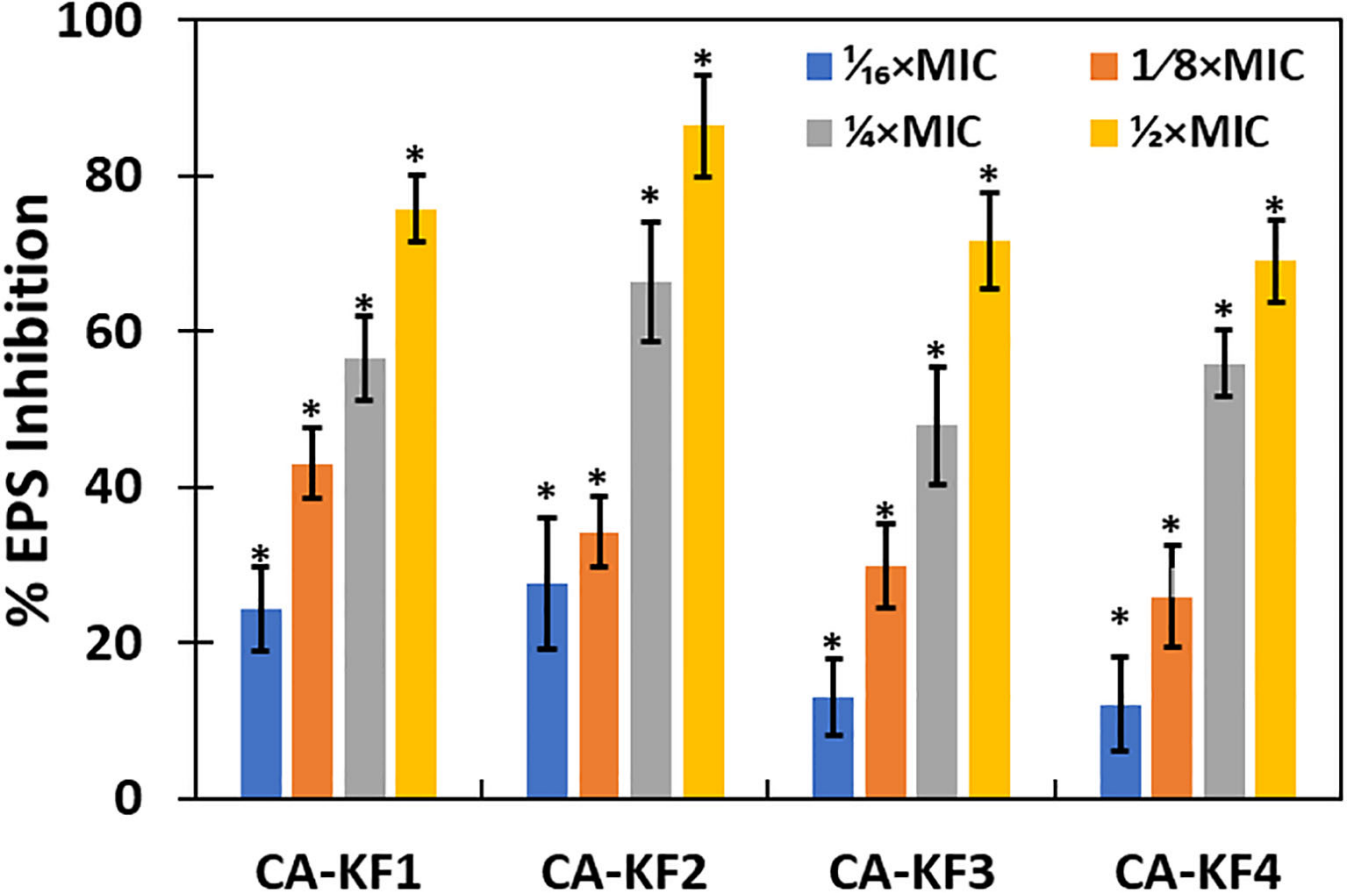
Inhibition of EPS production in AvTO-NPs-treated cells. Data are presented as average of three replicates and error bars depicting standard deviation. * denotes significance p ≥ 0.05.

The influence of sub-MICs of AvTO-NPs on EPS production by the test strains of C. albicans was examined considering the crucial role that it plays in the development and maintenance of biofilm. The concentration-dependent effect was observed, meaning that with increase in the concentration of AvTO-NPs, the EPS inhibition also increased as shown in **Figure 10**. AvTO-NPs at 1/2xMICs reduced EPS production by 75.7%, 86.3%, 71.6%, and 69%, in CA-KF1, CA-KF2, CA-KF3, and CA-KF4, respectively.

EPS is crucial in the initial stage of the biofilm formation, as it mediates irreversible attachment of the cells to the surface. Furthermore, it helps in the maintenance of the biofilm by helping it to survive adverse environmental conditions and protect against the action of antimicrobial drugs and host immune responses. EPS prevents the cells from dehydrating, helps in ion exchange, stores and maintains degradation enzymes, and transports nutrients (Velayutham et al., 2012). Reduced production of EPS under the effect of sub-MICs of AvTO-NPs will not only expose the cells to the action of NPs but also render them susceptible to antifungals and human leukocytes.

### 3.11 Effect on established matured biofilms

Increased resistance of C. albicans towards antifungals in biofilm mode due to the expression of some genes encoding for resistance and certain phenotypic modifications is well documented. Indeed, increased resistance towards fluconazole by C. albicans biofilms have been reported previously (Uppuluri et al., 2011). Therefore, disturbance and disruption of established matured biofilms is an attractive proposition in the development of effective antifungals for the treatment of persistent infections like DFUs.

**Figure 11:**
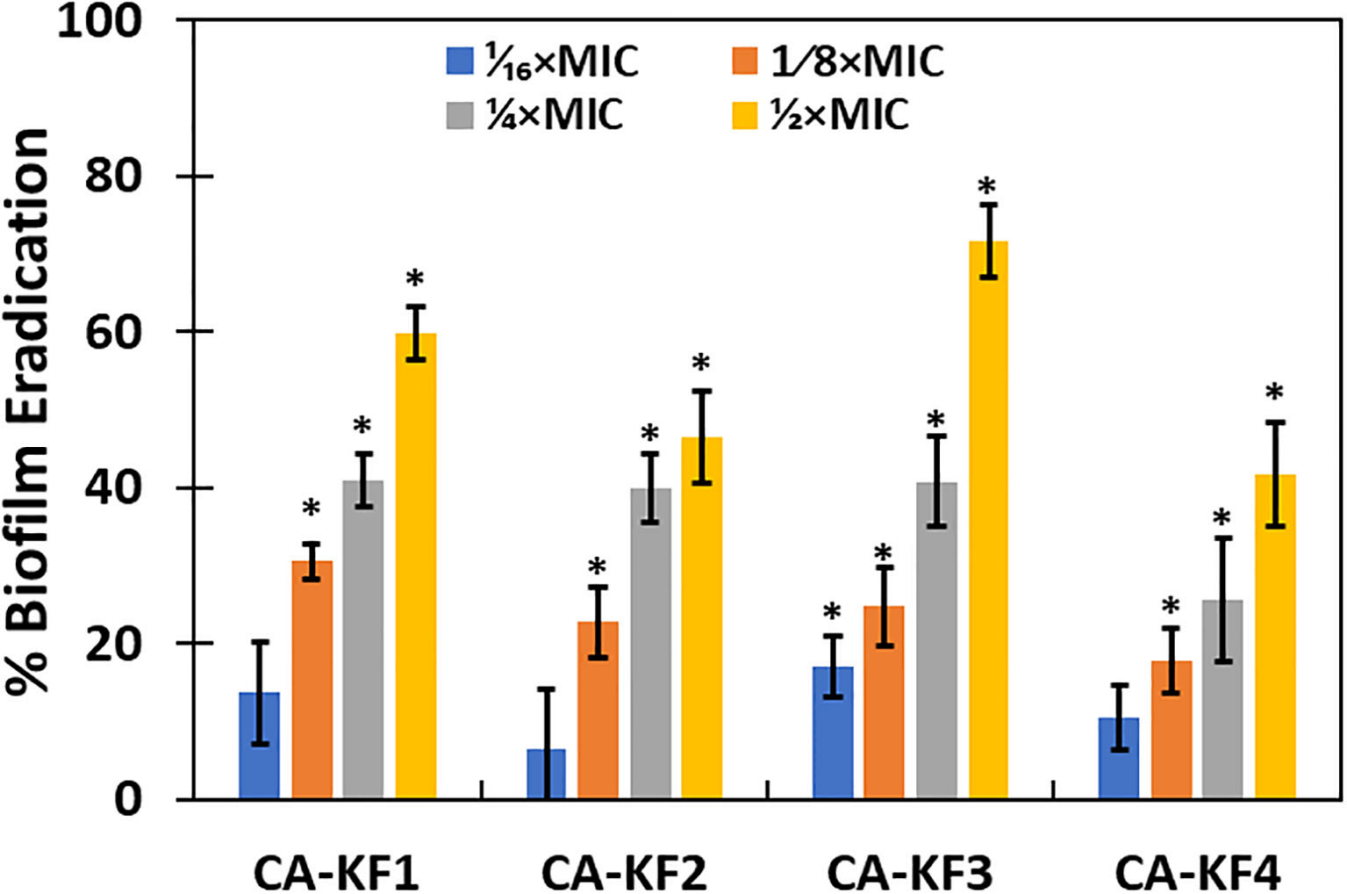
Eradication of pre-formed biofilms by sub-MICs of AvTO-NPs. Data are presented as average of three replicates and error bars depicting standard deviation. * denotes significance p ≥ 0.05.

In the present study, 1/16xMICs–1/2xMICs of AvTO-NPs were used to assess their effect on preformed biofilms of C. albicans strain. **Figure 11** shows significant (p< 0.05) reduction in mature biofilms in all test pathogenic strains at concentrations ranging from 1/8xMIC to 1/2xMIC. Since the inhibition observed was concentration dependent, maximum reduction of 59.7%, 46.3%, 71.6%, and 41.6% in CA-KF1, CA-KF2, CA-KF3, and CA-KF4, respectively, was recorded at highest tested concentration (1/2xMIC). Overall, AvTO-NPs were effective in disrupting mature biofilms of test strains significantly at respective sub-MICs. Our findings corroborate well with the effect demonstrated by silver nanoparticles (AgNPs) and selenium nanoparticle (SeNPs) against preformed biofilms of C. albicans. Dose-dependent inhibitory effect of AgNPs and SeNPs on preformed biofilms of C. albicans was recorded, with a calculated IC_50_ of 0.089 ppm and 21.7 ppm, respectively (Lara et al., 2015, Lara et al., 2018).

### 3.12 ROS-mediated biofilm inhibition

**Figure 12:**
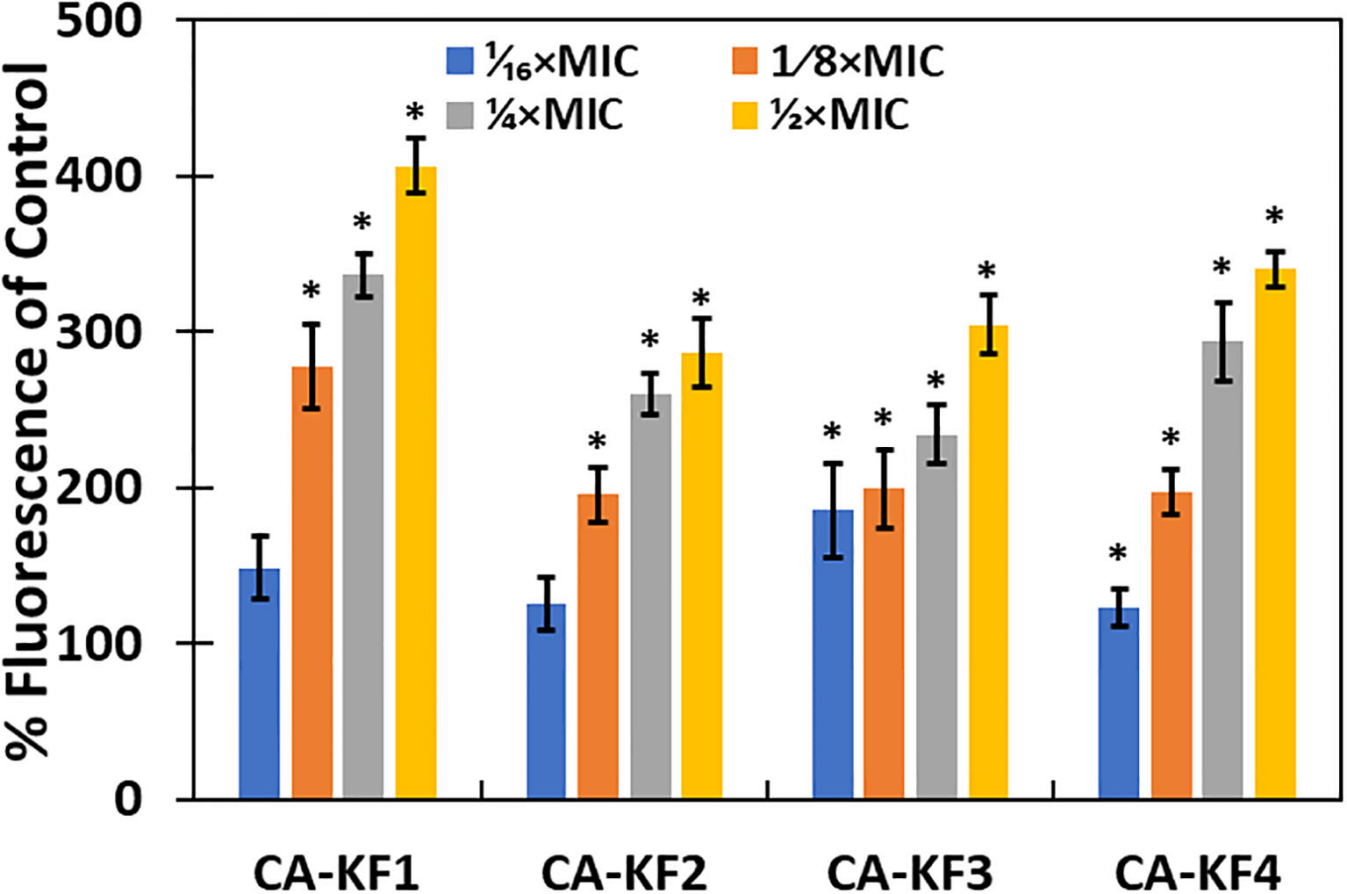
ROS generation in *C. albicans* treated with or without AvTO-NPs. Data are presented as average of three replicates and error bars depicting standard deviation. * denotes significance p ≥ 0.05.

Intracellular ROS generation in NPs-treated cells has been identified as a mechanism for the biofilm inhibition in microbial cells (Dwivedi et al., 2014). The relative amount of intracellular ROS generated in AvTO-NPs-treated C. albicans strains was examined using fluorescent probe DCHF-DA. As evident from **Figure 12**, significantly elevated ROS levels were recorded in test strains treated with sub-MICs of AvTO-NPs. In the presence of 1/2xMICs of NPs, C. albicans strains CA-KF1, CA-KF2, CA-KF3, and CA-KF4 showed 406.4%, 286.1%, 304.5%, and 340% upsurge in the ROS levels, respectively, as compared to untreated cells. ROS-scavenging system exists in microbial cells to counter-balance the ROS generated under non-stress environments. Under stress, this ROS production increases to such an extent that the ROS-scavenging enzymes are outclassed and rendered ineffective. This failure of the antioxidant defense machinery to scavenge generated ROS leads to oxidative stress damaging DNA, proteins, lipids, and eventually cell death (Zubair et al., 2021). It is envisaged that the AvTO-NPs-induced increased ROS generation could be responsible for the death of C. albicans cells residing in the biofilm mode.

### 3.13 Effect on renal function markers (creatinine and urea)

**Figure 13:**
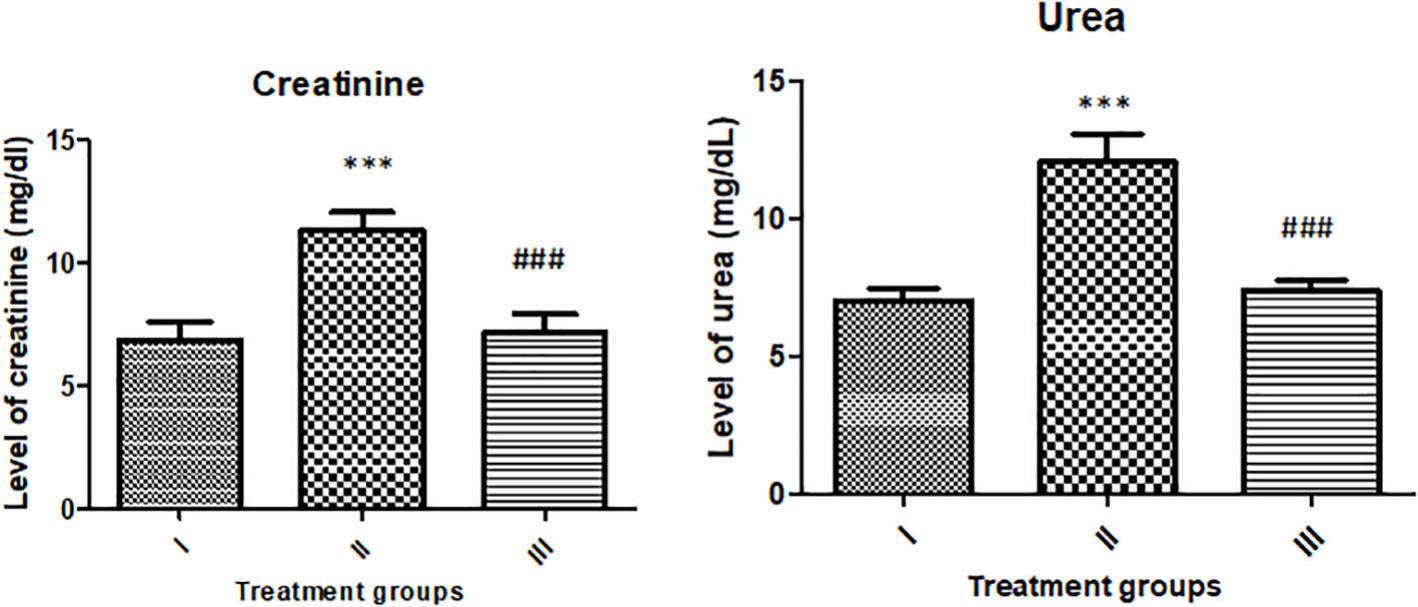
Showing the effect of the treatment on renal function markers (urea and creatinine). All the data expressed as mean ± SD for each group (n=6). *** indicates statistical significance from control (group I), while ### indicates significant difference from group II.

Groups II and III showed elevated creatinine levels by 65.23% and 4.70% with respect to the control, group I. The level of urea in groups II and III was increased by 72% and 5.35% with respect to the control (**Figure 13**).

### 3.14 Effect on liver function markers (AST and ALT)

**Figure 14:**
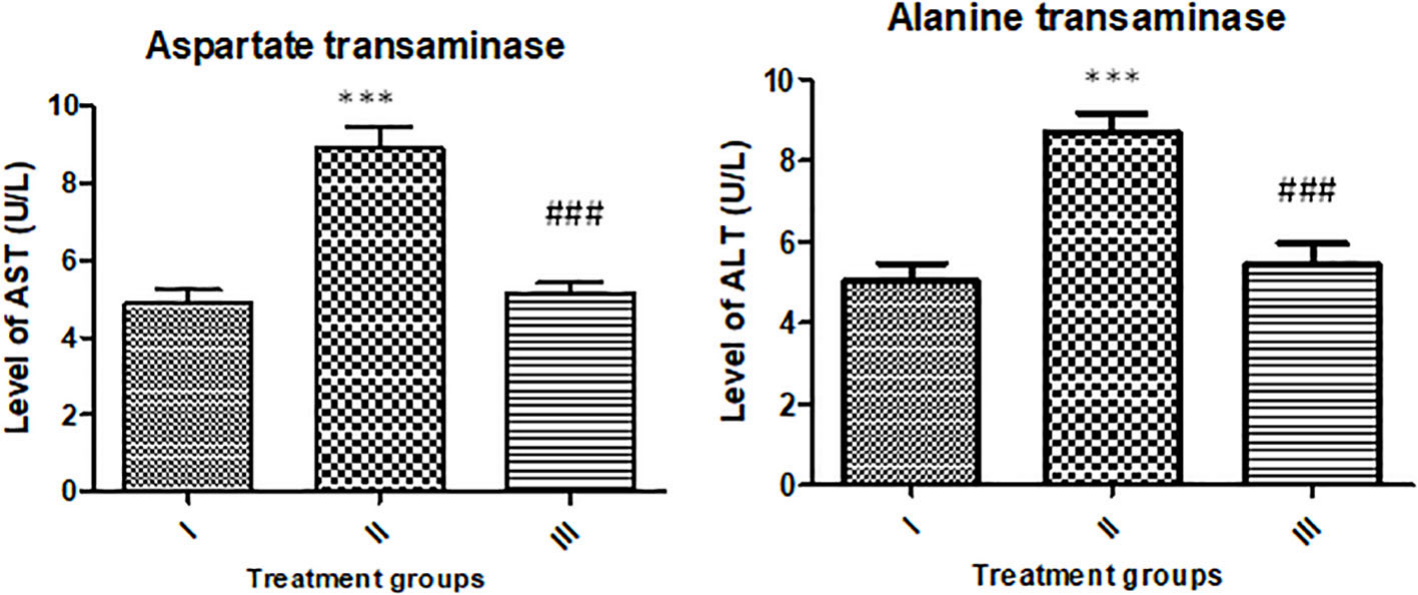
Showing effect of the treatment on liver function markers (AST and ALT). All the data expressed as mean ± SD for each group (n=6). *** indicates statistical significance from the control (group I), while ### indicates significant difference from group II.

The activity of AST was enhanced by 83.61% in group II, while group III showed an increase in its activity by 5.40% compared to the control. However, the activity of ALT was found to be enhanced by 73.33% and 8.05% with respect to the control (**Figure 14**).

The results of the renal and liver function markers in the serum samples indicate that the reported nanoparticles exert no significant toxic burden on the major target organs—liver and kidneys. Similar results were also reported in the recent studies by Alhazza et al, 2023. Therefore, the in vivo study confirms that the nanoparticles pose no major toxicity towards the animals; hence, the NPs are safe to the animals to conduct further investigations. The current findings are results from a short study on animal model. Further long-term and in-depth in vivo evaluation is warranted before the clinical usage of the nanoparticles.

## 4 Conclusions

The current investigation reports the synthesis of tin oxide (SnO_2_) NPs using A. vulgaris extract as a stabilizing agent for the first time. AvTO-NPs were effective in inhibiting biofilm formation and mature established biofilms of pathogenic C. albicans strains significantly. Since C. albicans biofilms show resistance towards fluconazole, disruption of established matured biofilms by AvTO-NPs is an interesting finding. Moreover, AvTO-NPs demonstrated antivirulence activity by significantly reducing the formation of the germ tube, EPS production, and cell surface hydrophobicity of C. albicans. These virulence factors greatly contribute in the pathogenicity of C. albicans and are responsible for the formation of drug-resistant biofilms. We have established enhanced intracellular ROS production in NPs-treated cells as the plausible mechanism of biofilm inhibition. In summary, we have highlighted the potential of biosynthesized NPs in mitigating the threat of drug-resistant pathogens of clinical origin colonizing the wounds such as DFUs. These findings could be a starting point in the development of novel therapeutics targeting C. albicans biofilms. Furthermore, these NPs can be utilized in combinational therapies to target drug-resistant pathogens and develop a paradigm that expedites healing of wounds, limits spread of infections, and eventually reduces the risk of amputations. Furthermore, we need to study the effect on in vivo biofilms in animal model systems, and molecular mechanisms of biofilm inhibition need to be uncovered.

## Data availability statement

The original contributions presented in the study are included in the article/**Supplementary Material**. Further inquiries can be directed to the corresponding author.

## Ethics statement

This study was approved by the Local Research Ethics Committee (LREC) of the University of Tabuk, Tabuk. KSA vide its approval no. UT-191-59-2022 under the aegis of National Committee of Bioethics (NCBE) of Kingdom of Saudi Arabia and informed consent was obtained.

## Author contributions

MZ: Conceptualization, Funding acquisition, Writing – original draft, Writing – review & editing. FH: Conceptualization, Formal Analysis, Methodology, Writing – original draft, Writing – review & editing. MAl: Investigation, Validation, Writing – review & editing. ImH: Investigation, Writing – review & editing. IfH: Formal Analysis, Investigation, Writing – original draft. TA: Software, Visualization, Writing – original draft. FF: Conceptualization, Formal Analysis, Writing – review & editing. AK: Formal Analysis, Methodology, Writing – review & editing. Mar: Conceptualization, Visualization, Writing – review & editing. PA: Conceptualization, Formal Analysis, Writing – original draft. NA: Investigation, Validation, Writing – original draft. RA: Validation, Visualization, Writing – review & editing. SB: Investigation, Resources, Writing – review & editing. RM: Data curation, Validation, Writing – review & editing. HA: Software, Data curation, Writing – original draft. AA: Writing – review & editing, Resources, Software. A-fA-A: Data curation, Methodology, Writing – review & editing.
