# Supplementary material for: In vitro inhibition of biofilm and virulence factor production in azole-resistant strains of Candida albicans isolated from diabetic foot by Artemisia vulgaris stabilized tin (IV) oxide nanoparticles
Source: Front Cell Infect Microbiol. 2024 Jan 25;13:1322778. doi: 10.3389/fcimb.2023.1322778 (PMC10850385; doi:10.3389/fcimb.2023.1322778)
Supplement: Supplementary file 1 [file Table_1.docx]

**Supplementary file**

**In vitro inhibition of biofilm and virulence factor production in Azole-resistant strains of albicans and non-albicans *Candida* isolated from diabetic foots by phyto-fabricated tin oxide nanoparticles**

|  | | **Amphotericin B (10µg)** | **Itraconazole (10µg)** | **Fluconazole (10µg)** | **Ketoconazole (10µg)** |
| --- | --- | --- | --- | --- | --- |
| *C. albicans*  *N=28* | Resistance n(%) | 12 (42.8) | 6 (21.4) | 4 (14.2) | 5 (17.8) |
|  | Sensitive n(%) | 16 (57.1) | 22 (78.5) | 24(85.7) | 23 (82.1) |

**Table S1. Antifungal Activity**

**Table S2. Biofilm Activity**

|  | **Biofilm producers** | | | **Non-Biofilm producer** |
| --- | --- | --- | --- | --- |
| *C. albicans*  *N=28* | 18 (64.2) | Strong | 4(22.2) | 10 (35.7) |
|  |  | Intermediate | 9 (50.0) |  |
|  |  | Week | 5 (27.7) |  |
